# Antibody-Electroactive Probe Conjugates Based Electrochemical Immunosensors

**DOI:** 10.3390/s20072014

**Published:** 2020-04-03

**Authors:** Mateusz Kondzior, Iwona Grabowska

**Affiliations:** Institute of Animal Reproduction and Food Research, Polish Academy of Sciences, Tuwima 10, 10-748 Olsztyn, Poland; mateuszkondziorr@gmail.com

**Keywords:** immunosensors, electrochemistry, conjugation of antibodies, redox active probes, nanomaterials, simultaneous multi-analyte detection

## Abstract

Suitable immobilization of a biorecognition element, such as an antigen or antibody, on a transducer surface is essential for development of sensitive and analytically reliable immunosensors. In this review, we report on (1) methods of antibody prefunctionalization using electroactive probes, (2) methods for immobilization of such conjugates on the surfaces of electrodes in electrochemical immunosensor construction and (3) the use of antibody-electroactive probe conjugates as bioreceptors and sensor signal generators. We focus on different strategies of antibody functionalization using the redox active probes ferrocene (Fc), anthraquinone (AQ), thionine (Thi), cobalt(III) bipyridine (Co(bpy)_3_^3+^), Ru(bpy)_3_^2+^ and horseradish peroxidase (HRP). In addition, new possibilities for antibody functionalization based on bioconjugation techniques are presented. We discuss strategies of specific, quantitative antigen detection based on (i) a sandwich format and (ii) a direct signal generation scheme. Further, the integration of different nanomaterials in the construction of these immunosensors is presented. Lastly, we report the use of a redox probe strategy in multiplexed analyte detection.

## 1. Introduction

Electrochemical immunosensors are a type of integrated devices that provide selective quantitative or semi-quantitative analytical information using biorecognition phenomenon between an antibody (Ab) and antigen (Ag) with an electrochemical transducer [1]. Due to the stable, strong and specific binding between these biomolecules, electrochemical immunosensors are characterized by high selectivity and sensitivity [2]. These analytical devices have found application in different fields, including food, environmental, agricultural analysis, clinical diagnosis and others [3,4,5]. 

The interactions between Ab and Ag can be observed using different labels, such as radioactive, chemiluminescent and fluorophore compounds. Enzymes are another group of labels, including horseradish peroxidase (HRP), alkaline phosphatase (ALP), laccase and glucose oxidase (GOx), which need some substrates added to the testing solution, such as hydroquinone, catechol, o-aminophenol, naphthyl phosphate, p-aminophenol phosphate, ferrocene and glucose [3]. 

In the past few years, the enzyme-linked immunosorbent assay (ELISA) used for specific detection of different analytes has become very popular and commonly used in laboratory practice. This method, depending on the format of the assay (i.e., direct, sandwich or competitive) could be equally useful for the detection of both antigens and antibodies. It uses the secondary antibodies conjugated with a specific enzyme. After addition of a suitable substrate, the enzyme catalyzes a specific reaction, and the product can be quantified spectrophotometrically to measure the color intensity. Thus, in fact, ELISA is an optical approach, which has some disadvantages, such as requirements related to light sources, detectors and monochromators, specified sample volume and the length of the optical path, as well as the occurrence of false signals arising from complex colored samples [2]. Apart from undeniable positives, such as high sensitivity and specificity, these tests are characterized by time-consuming analyses consisting of several washing steps. Moreover, low thermal and environmental stability due to the denaturation, sensitivity of catalytic activity to environment condition, non-conductivity, difficult and expensive synthesis and purification of enzymes are the other drawbacks. Wherefore, the enzymatic labels were recently substituted by different nanoparticles, including gold nanoparticles, silver nanoparticles, quantum dots, cerium oxide nanoparticles, mercury selenide nanoparticles and copper-based nanoparticles [6,7]. In recent years, electrochemical ELISA-based immunosensors have also become very popular [8]. This system combines the advantages of optical ELISA and electrochemical methods. Compared with traditional optical immunoassays, electrochemical immunosensors are characterized by simplicity, speed, low cost, portable and easy to use instrumentation, greater possibilities of miniaturization and continuous monitoring in real-time, as well as usability for multiplexing [4]. 

There are two types of labels used in immunosensors based on electrochemical detection: (1) a label that is electroactive, (2) an enzyme label that catalyzes the production of an electroactive product. Application of electroactive labels instead of enzyme labels has created an attractive alternative due to the simplified protocol, wider linear range and higher stability [9]. However, the distinct advantage of enzyme labels over other labels has been connected with sensitivity based on the “multification effect”. The enzyme label has been used for detection by monitoring the product in the presence of substrate. The accumulation of product has appeared, which is generated by the enzyme far more than a stoichiometric amount. The possible solution for increasing the sensitivity of immunosensors based on electroactive labels as compared with immunosensors based on enzyme labels is increasing the number of redox active molecules attached to antibodies. This issue will be more deeply discussed in the next section.

**Figure 1:**
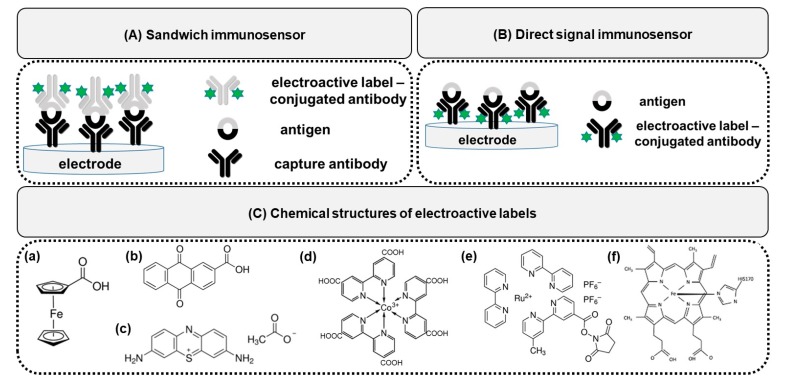
General scheme of immunosensor based on electroactive label conjugates: (**A**) in sandwich format; (**B**) in direct signal format; (**C**) chemical structures of different electroactive labels widely used for antibody bioconjugation: (**a**) ferrocenecarboxylic acid, (**b**) anthraquion-one 2-carboxylic acid, (**c**) thionine, (**d**) tris(2,2′-bipyridine-4,4′-dicarboxylic acid)cobalt(III), (**e**) tris(bipyridine)ruthenium(II) with N-succinimidyl ester group, (**f**) iron heme group in horseradish peroxidase.

In this review, we are going to concentrate on the antibody-electroactive probe conjugates and their use in sandwich and direct signal immunosensors. A sandwich-type-format immunosensor consists of an unlabelled primary capture antibody and an electrochemically-detectable, redox-active label-conjugated signalling secondary antibody (Figure 1A). Upon binding of a specific antigen and the next signalling antibody, an increase in the redox-active molecule current is detected. In the case of a direct signal immunosensor, the antibodies labelled with redox-active tags act as a capture and signalling antibody simultaneously, and the direct electron transfer of the label is detected (Figure 1B). Upon formation of immunocomplexes, the redox active label current decreases due to the increasing spatial blocking. 

The possible candidate of redox species to be labelled to antibodies should display several key issues, including: (I) be electroactive in an appropriate potential window, (II) be stable in buffer used in electrochemical studies, (III) not cause electrode fouling, (IV) possess some chemical groups available for coupling, (V) be easily integrated with bioactive molecules in simply way, (VI) possess good compatibility [3]. Among many possible candidates, ferrocene derivatives have found considerable interest for derivatization of biomolecules [10,11,12,13,14], mainly due to the reversible redox characteristic being in a suitable potential range [15]. Due to the low cost and efficient electron transfer, the following redox active labels have been used in biological systems: phenazine dye, neutral red, toluidine blue, Prussian blue, methylene blue, azure A and thionine. The other small and stable molecule easily introduced to any biomolecules is anthraquinone. The other label worth mentioning is tris(bipyridine)ruthenium(II) [Ru(bpy)_3_]^2+^, which combines the possibilities of electrochemical detection with electrochemiluminescence (ECL) [16,17].

The chemical structures of the most frequently used redox active labels in conjugation with antibodies are presented in Figure 1C.

The other important issue related to immunosensors is the proper immobilization of antibodies on the surface of electrodes, which affects the sensitivity and specificity of antigen determination. There are different methods of antibody immobilization on the surface of electrodes in immunosensors, including irreversible and reversible methods [18]. The covalent binding between an antibody and the solid support is one among the most widely used. Just for examples, immobilization of antibodies on gold surface modified with 3,3′-dithiobis (sulfosuccinimidyl) propionate (DTSSP) [19] or immobilization of thiolated antibodies [20] has been reported. The immobilization of antibodies on the protein G layer allows for their orientation with the Fc domain bound to the protein G layer, and the Fab domains face away from the surface [21]. 

In the construction of high-performance immunosensors, the use of different nanomaterials, e.g., carbon nanotube, graphene, indium tin oxide, nanowire and metallic nanoparticles has been recently reviewed [22]. The latest progress in the area of electrochemical immunosensors has been related to the modification of working electrode surfaces using novel nanomaterials in order to obtain superior sensor sensitivity [23]. Significantly low detection limits could be obtained using nanoelectrode ensembles (NEEs) [24,25,26,27]. These electrodes are characterized by a detection limit of 2–3 orders magnitude lower than a macroelectrode with the same geometric area. This is because of the high ratio between faradaic and double layer charging currents, which is reflected in very high S/N ratios.

One of the many remaining challenges in electrochemical immunosensor development concerns simultaneous multianalytes detection. This is extremely important in the context of selective and sensitive detection of biomarkers, which plays a crucial role in disease diagnosis, treatment and monitoring of its progress [28]. In clinical practice, the detection of a single biomarker is usually not sufficient, and the detection of several biomarkers is an effective solution in improving diagnostic value. Recently, the interest in simultaneous multianalyte-based immunosensors has attracted more attention than ever [29], and this issue will also be highlighted in this review.

## 2. Procedures for Antibody Conjugation with Electroactive Probes

Methods based on precise and site-selective modification of antibodies, for application mainly in the medicine sector, have been developed for few last years [30,31]. Antibody drug conjugates (ADCs) are an important class of drugs that combine the specificity of monoclonal antibodies (mAbs) with the potent cancer killing ability of small molecules [32]. The ADCs are three component molecules composed of drug or payload, linker and an antibody, most often immunoglobulins G (IgGs). IgGs possess natural amino acids in the structure such as lysine or cysteine, which are attractive sites for conjugation. This class of antibodies consists of 80 lysines, of which more than 20 are characterized by solvent-accessible lysine residues able to induce amide coupling between amine groups and activated carboxylic ester. These –NH_2_ groups are distributed irregularly across the whole molecule of the antibody. Most of the procedures described below are based on the conjugation of labels to the –NH_2_ groups derived from lysines.

**Figure 2:**
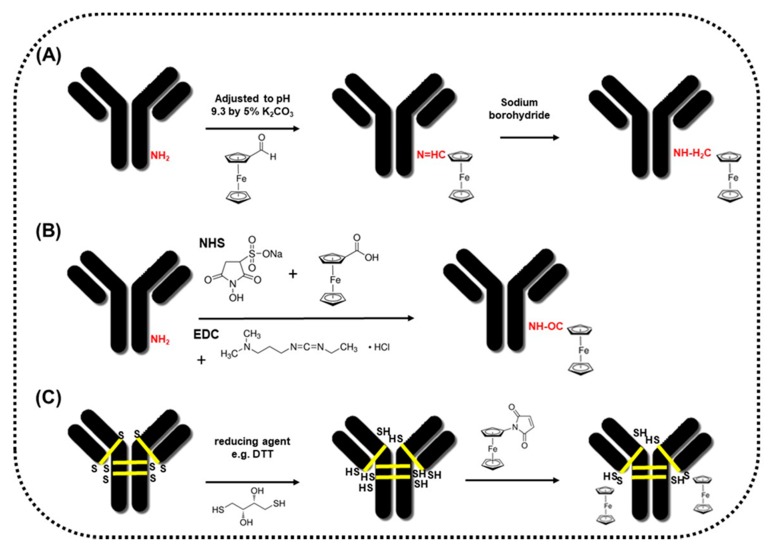
Schematic representation of procedures for antibody labelling with (**A**) ferrocenecarboaldehyde, (**B**) ferrocenemonocarboxylic acid, (**C**) ferrocenemaleimide.

The method of antibody modification with ferrocene using mixed anhydride activation of ferrocenecarboxylic acid was presented by Kossek and co-workers in 1996 [33]. The activation of ferrocenecarboxylic acid was done by addition of tributylamine and later isobutyl chloroformate. The coupling of activated ferrocene with the antibody took 2 h at room temperature. The final product was dialyzed against phosphate buffer. The same activation method has been used for antibody (Ab) conjugation with ferrocene (Fc) with various ratios of both components Ab:Fc [34]. The authors confirmed the redox activity of various antibody-ferrocene conjugates using cyclic voltammetry (CV) studies with a carbon paste microelectrode. They observed increased ferrocene electroactivity with the increasing of its moiety in the cases of conjugates with a molar ratio of Ab:Fc: 1:3.8, 1:4.6, 1:19.3, 1:35.4 and 1:125.8, respectively. Whereas, immunoreactivity between the antigen (here digoxin) and its antibody did not change for all conjugates up to a 1:35 molar ratio of Ab:Fc and dropped by 50% for conjugates with a molar ratio of 1:125.8. In the procedure described by Lim and Matsunaga [35], the mixture of ferrocenemonocarboxylic acid, EDC (1-ethyl-3-(dimethylaminopropyl) carbodiimide hydrochloride and IgG was incubated for 4 h at room temperature. Atomic absorption spectroscopy experiments showed that 11 iron atoms from ferrocene were conjugated to IgG, whereas cyclic voltammetry experiments proved the electrochemical properties of ferrocene conjugated with IgG at the graphite electrode. The oxidation of ferrocene in Fc-IgG immunocomplex has been observed at a potential of 395 mV versus Ag/AgCl. Similarly, as in the previous papers, authors have observed the oxidative current increasing with relation to an increasing number of ferrocene molecules on the IgG antibody. A comparison of two methods of antibody conjugation has been published by Okochi and others [36]. The first procedure presented in Figure 2A involves modification of IgG with ferrocenecarboaldehyde (Fc-CHO) via formation of a Schiff base and its reduction using sodium borohydride. The second protocol (Figure 2B) was based on a conventional method using activation of the –COOH group from ferrocenemonocarboxylic acid (Fc-COOH) using EDC, followed by addition of NHS (N-hydroxysuccinimide) in order to increase efficiency and creation of dry-stable intermediates. The authors showed that there is a three-fold improvement in the efficiency of the first procedure based on Fc-CHO as compared to the second procedure based on Fc-COOH. Authors have estimated eight ferrocene moieties labelled to IgG using the Fc-CHO method and atomic absorption spectroscopy experiments. Electrochemical signals for IgG labelled with Fc-CHO increased by 14-fold as compared to IgG labelled with Fc-COOH, as observed at the potential of 390 mV vs. Ag/AgCl. Thus, it has been proved that the number of redox-active moieties on IgG influences sensitive detection of particular antibodies. Despite those results, the preparation of antibodies labelled with Fc-COOH has been frequently reported in many papers [37,38].

In contrast to lysines, cysteines are present in antibodies in a lesser quantity, but they are uniformly located. There are 16 disulphide bonds in the IgG molecule: 12 intra-chain and 4 inter-chain. The latter are the main targets for conjugation, because of greater solvent accessibility. Before conjugation, the disulphide bonds should be carefully reduced to thiols, mostly using reducing agents, such as dithiothreitol (DTT) or tris(2-carboxyethyl)phosphine (TCEP) [39]. The derivatives of compounds possessing the maleimide group could react with these thiols according the schematic illustration presented in Figure 2C. The conjugation to the reduced disulphide bonds, which are present in the hinge region of the antibodies, away from the antigen binding sites could potentially minimize any loss of antibody-antigen binding activity [40]. Even the most careful optimization of conjugation conditions could potentially lead to low conjugation efficiency (low redox active molecule to antibody ratio). Moreover, some antibodies are prone to aggregation or the product of conjugation could precipitate. 

The procedures presented above have been conducted in solution and have several limitations including the need for purified antibodies at high concentration and a lot of steps for buffer exchange. A method based on-bead conjugation has appeared that is suitable for antibody purification and conjugation in a single workflow [41].

The proper labelling of antibodies with redox active molecules guarantees their adequate deposition on the surface of gold electrodes. We suggest application of ADC technology based on the reduction of disulphides from cysteines for the attachment of maleimide derivatives of redox active molecules, as for example ferrocene or other compounds. Such modification will allow the labelled antibodies to be attached to the surface of gold electrodes, mainly because of the presence of the free, unmodified Fc fragment of the antibody.

## 3. Sandwich Immunosensors Based on Antibody-Electroactive Probe Conjugates

**Figure 3:**
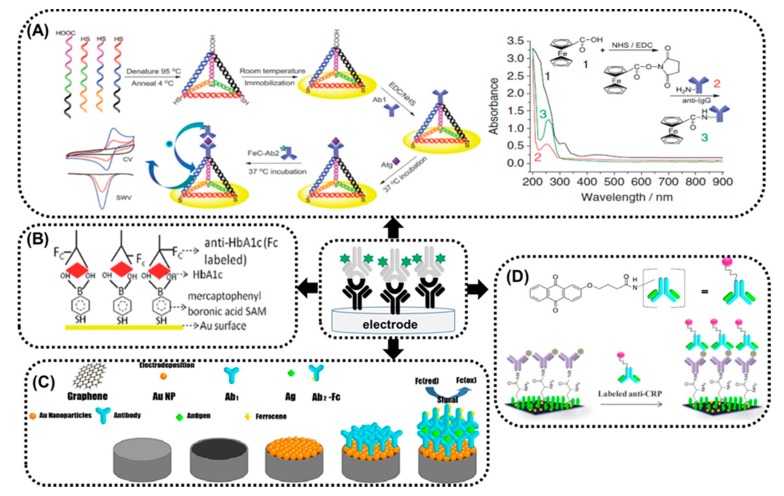
Selected examples of sandwich immunosensors based on electroactive label conjugates. (**A**) Schematic illustration of Au electrode modification using the DNA nano-pyramids (DPs) and ferrocene-labelled anti-IgG and Uv-vis spectra of ferrocenecarboxylic acid and anti-IgG, FeC-anti-IgG (reprinted with permission from Yuan et al. [43]). (**B**) Scheme of gold screen-printed sensor modification using mercaptophenyl boronic acid self-assembled monolayers and ferrocene-tagged anti-glycated haemoglobin antibodies (reprinted with permission from Chopra et al. [44]). (**C**) Scheme of the fabrication of the sandwich immunosensor based on graphene/gold nanoparticle composite and ferrocene-labelled secondary antibodies (reprinted with permission from Wang et al. [45]). (**D**) Schematic illustration of screen-printed graphene electrode modification using anthraquinone-labelled, C-reactive, protein-specific antibodies (reprinted with permission from Jampasa et al. [46].

There are a number of different examples of amperometric immunosensors based on antibody-electroactive probe conjugates in sandwich format available in the literature [42]. The scheme for the molecular construction of this type of sensor contains the capture antibody able to bind antigen and electroactive label-conjugated antibodies that recognize different epitopes of antigens. The change in the oxidation/reduction of this label is used for quantitative determination of the antigen. Most of published immunosensors differ in the method of capture for antibody immobilization on the solid surface of an electrode and the type of label conjugated to secondary antibodies. Among others, thanks to fast and reversible redox reactions, ferrocene is the most frequently used for conjugation to antibodies. The following examples of sandwich type amperometric immunosensors will be presented herein (Figure 3).

In the first very elegant example of a sandwich immunosensor [43], DNA nano-pyramids (DPs) were presented to be anchored to a gold electrode surface and serve as a base for attachment of capture anti-IgG antibodies via a free-standing carboxyl group present at the top vertex (Figure 3A). Ferrocene-based anti-IgG has generated significant electrochemical responses upon complexation of immunoglobulin G (IgG). DPs, thanks to their elegant three-dimensional (3D) structure, reduce the local overcrowding effect, facilitate better orientation and accessibility for antibody coupling and hence improve the formation of the sandwich complex, as well as minimize nonspecific adsorption. Moreover, the pyramid’s hollow structure supports electron transfer between ferrocene and the surface of the electrode, which leads to ultrasensitive detection of IgG. Authors speculated that, in their system, the charge transfer mechanism is based on the assumption that ferrocene moiety in the Ab1-Atg-(FeC-Ab2) complex is flexible enough to physically impinge on the electrode surface and maintain a sufficiently high current signal. Moreover, the immunosensor presented was highly reproducible, as proved using intra-assay and inter-assay precision displaying relative s. ds. of 9.23% and 8.55%, respectively.

In another study [44], the derivative of boronic acid self-assembled onto a gold screen-printed electrode was proposed as a capture for glycated haemoglobin via creation of diol linkages (Figure 3B). Anti-glycated haemoglobin antibody tagged with electroactive ferrocene has been used as detector molecules in a sandwich immunoassay format. Such a novel combination has improved the specificity and signal response towards glycated haemoglobin. 

The need for improvement in electrochemical immunosensor parameters has turned scientists’ attention to various novel materials. The combination of graphene with gold nanoparticles has been used by Wang and co-workers [45] to construct a novel sandwich immunosensor based on ferrocene derivatives as labels for the detection of IgG (Figure 3C). The electrochemical behaviour of three ferrocene derivatives used as labels differing in structure has been studied, Fc_(1)_-ferrocenemonocarboxylic acid, Fc_(2)_-β-ferrocenyl-propenoicacid and Fc_(3)_-1,1′-ferrocenedicarboxylic acid. Fc_(3)_ appears to be the best label among the three due to the higher number of COOH-groups for labeling. This label has presented a high loading of antibody molecules and reserved higher immunological activity by the structure. The reproducibility of this sensor was satisfactory as presented by RSDs (relative standard deviations) of intra- and inter-assay, which displayed of values of 4.2%, 6.1%, 6.7% and 5.2%, 5.9% and 6.7% for 100, 200 and 300 ng/mL IgG, respectively.

Another strategy for signal amplification has been proposed by Sharma et al [47]. The bionanocomposite containing graphene-chitosan-gold nanoparticles capturing antibodies has been used for the construction of electrochemical sandwich immunoassay using ferrocene-labelled detection antibodies for the detection of Staphylococcus enterotoxin B (SEB). The signal amplification strategy based on electropolymerization of gold electrodes with o-phenylenediamine and further attachment of cysteine with glutaraldehyde as a cross-linker and gold nanoparticles has been proposed by Zhang and co-workers [48]. Such a prepared sensing interface was used for anti-IgG antibody adsorption and sandwich complex formation with IgG and ferrocene-labelled anti-IgG. In another example, Akram and co-workers [49] describe a method of glassy carbon electrode pre-treatment in order to get -COOH groups on its surface to employ carbodiimide reaction to attach a capture antibody specific for human chorionic gonadotropin (hCG). Direct oxidation of ferrocene conjugated to the signal antibody observed using cyclic voltammetry showed a linear change of peak current density in relation to different concentrations of hCG.

Except ferrocene, the use of anthraquinone (AQ)-labelled signalling antibody in a sandwich-type-format immunosensor has been recently proposed [46]. The electrochemical signal of AQ antibody has been measured using differential pulse voltammetry (DPV) upon binding of the C-reactive protein (CRP) to the primary antibody covalently anchored onto a screen-printed graphene electrode (Figure 3D). Increasing concentration of CRP caused an increase of current, observed in a concentration-dependent manner. Anthraquinone could be proposed as a good alternative redox label for introduction to any biomolecule thanks to its stability and small size, which minimizes the interference with the biomolecular interactions. Excellent fabrication reproducibility was obtained, expressed as a RSD lower than 5%.

The examples presented above show how the designed interface between the biocomponent and the detector and signal amplification strategies have been improved over the years.

## 4. Direct Signal Immunosensors Based on Antibody-Electroactive Probe Conjugates

**Figure 4:**
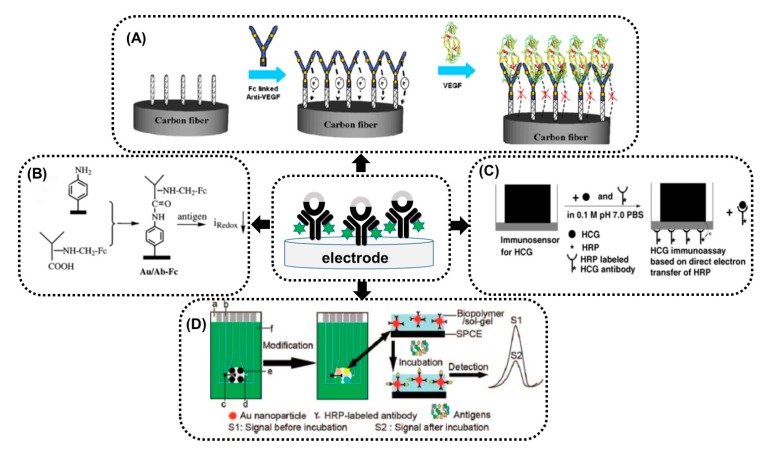
Selected examples of direct signal immunosensors based on electroactive label conjugates. (**A**) Scheme representing the construction of an immunosensor based on ferrocene monocarboxylic acid-tagged anti-VEGF antibodies immobilized on carbon fibre microelectrodes using Jeffamine cross-linker (reprinted with permission from Prabhulkar et al. [50]). (**B**) Procedure of immunosensor fabrication based on a polyaniline-modified gold surface employing covalently-bound ferrocene-labelled antibodies (reprinted with permission from Dou et al. [51]. (**C**) Scheme of immunosensor based on a glassy carbon electrode modified with titania sol-gel and horseradish peroxidase (HRP)-labelled human serum chorionic gonadotrophin (HCG) antibody (reprinted with permission from Chen et al. [52]). (**D**) Schematic illustration of immunosensor array based on a biopolymer/sol-gel membrane and HRP-labelled antibody-modified gold nanoparticles (reprinted with permission from Wu et al. [53].

Immunosensors based on a direct format include electroactive label-conjugated antibodies, which serve as a biological recognition element and signal conversion unit at the same time. There are a few examples of amperometric immunosensors based on antibody-electroactive probe conjugates in direct signal format available in the literature (Figure 4). 

In example work presented by Prabhulkar and others [50], the reagentless and mediatorless strategy for the detection of cancer biomarker vascular endothelial factor (VEGF) has been described. The disc-shaped carbon fibre microelectrodes were fabricated and modified with ferrocene monocarboxylic acid labelled anti-VEGF antibodies using bifunctional polyetheramine cross-linker Jeffamine®ED-600 (Figure 4A). Such a procedure allowed for reagentless electrochemically-enhanced detection based on direct oxidation of the ferrocene label. The formation of immunocomplex between the ferrocene-labelled anti-VEGF antibody and the VEGF caused a decrease of the ferrocene oxidation peak current, registered at 0.52 V using linear sweep voltammetry in 0.5 M NaClO4 solution. The resultant drop in the oxidation current was further employed to quantify the amount of VEGF. The detection mechanism is connected with the increased spatial blocking caused by formation of bulky immunocomplexes, further hindering the electron transfer of the ferrocene molecules to the carbon fibre electrode surface. The presented detection strategy offers acceptable reproducibility indicated by a max value of the RDS of 8.9% for the intra-assay and 11.2% for the inter-assay.

The immobilization of ferrocene-prefunctionalized antibodies on a gold electrode surface modified with polyaniline formed based on electrochemical reduction of diazonium salt has been developed [51] (Figure 4B). The direct reduction/oxidation process of ferrocene with a potential at ca. 0.2 V occurring in PBS (phosphate-buffered saline) allowed for analysis of the cardiac biomarkers creatinine kinase (CK) and the human cytokine interleukin 10 (IL10). A decrease of signal intensity of the oxidation of ferrocene label when incubated with specific antigen was observed. Very recently, the thermodynamics and kinetics of human chorionic gonadotropin (hCG) binding to the surface of electrodes modified with ferrocene-tagged antibodies theoretically and experimentally have been studied [54]. Upon the formation of antigen-antibody complex, the blocking of ferrocene voltammetry occurs. Authors have proved that at a low antigen concentration, a Frumkin adsorption isotherm fits the data and repulsion between bound antigens plays a significant role. Studies have shown that the affinity constant between ferrocene-tagged antibodies and antigens is an order of magnitude larger in comparison with the case of untagged antibodies. This may suggest that the chemical hydrophobicity of the redox tag may support stronger binding between antigens and antibodies. The kinetic study has confirmed that this platform can be applied for short incubation of redox-tagged antibodies with antigens and quantitative immunoassays. 

Another electroactive label used as a signal probe upon conjugation to antibodies was iron in the heme group of horseradish peroxidase (HRP). HRP is one of the most widely used enzymes for analytical purposes and biosensors, because it is smaller, more stable and less expensive than other popular alternatives. Probably, the denaturation of proteins may result in the loss of the heme non-covalent group, the active site of HRP [55]. Unfortunately, problems have appeared related to the difficulties in observing the direct electrochemistry of the HRP label in immunoconjugates. A possible way to solve these problems is to use conductive composite materials to decrease the electron transfer impedance [52,56]. Initially, the preparation of reagentless and mediatorless immunosensors was proposed based on the direct electrochemistry of HRP labelled to an antibody and then antigen detection via a competitive mechanism [52,56,57]. Chen and co-workers [52] have shown the usefulness of titania sol-gel on a glassy carbon electrode for the construction of immunosensors for the detection of human chorionic gonadothropin (HCG) based on direct electrochemistry of HRP (Figure 4C). The observed decrease in the current of the immobilized HRP was linearly proportional to the concentration of HCG in incubation solution. This immunosensor has showed acceptable reproducibility with an estimated variation coefficient of intra-assay equal to 3.5% and inter-assay equal to 8.1%. Another extended strategy to construct the reagentless immunosensor was based on the encapsulation of HRP-labelled immunocomponent-adsorbed gold nanoparticles in titania sol-gel [56] or immobilized in designer organically-modified silicate (ormosil) sol-gel [58,59]. In order to increase the electrical communication between the redox sites of HRP and the sensing surface except for the Au nanoparticles, biopolymer chitosan in a sol-gel matrix formed on screen-printed carbon electrodes was applied [53]. This concept was used in the construction of reagentless immunosensor array for simultaneous detection of carcinoma antigens. In this work, the direct capture of HRP-labelled antibodies on working electrodes allowed for reduction/oxidation of iron from heme. The formation of complex between HRP-labelled antibodies and antigens caused blocking of electron transfer between the HRP and electrodes, and the signal registered before and after incubation with the antigen is proportional to the amount of immunocomplex, as illustrated in the schematic diagram (Figure 4D). In the next example [57], a glassy carbon electrode was modified with carcinoma antigen-125 (CA 125) and titania sol-gel using vapour deposition and further incubated with HRP-labelled CA 125 antibody. The differential pulse voltammetry displayed the reduction peak current of Fe(III) from HRP immobilized on the surface of the solid electrode at −0.558 V, which decreased with different concentrations of CA 125 due to the competitive binding of soluble CA 125 and immobilized CA 125 with the limited binding sites of the HRP-labelled CA 125 antibody.

## 5. Immunosensors Based on Multi-Antibody-Electroactive Probe Conjugates Strategy

The modern trends in the electrochemical immunosensors are based on the development of sensors for multiplexed determination of specific biomarkers [29,60,61]. The design of an immunosensor that is capable of simultaneous determination of two or more analytes in a single measurement, for example on a single working electrode in single solution, is a great challenge. Multi-analyte assays has many advantages compared to single analyte tests, such as cost per test, labour, throughput and convenience [62]. The following examples of immunosensors based on multi-antibody-electroactive probe conjugates will be presented herein.

**Figure 5:**
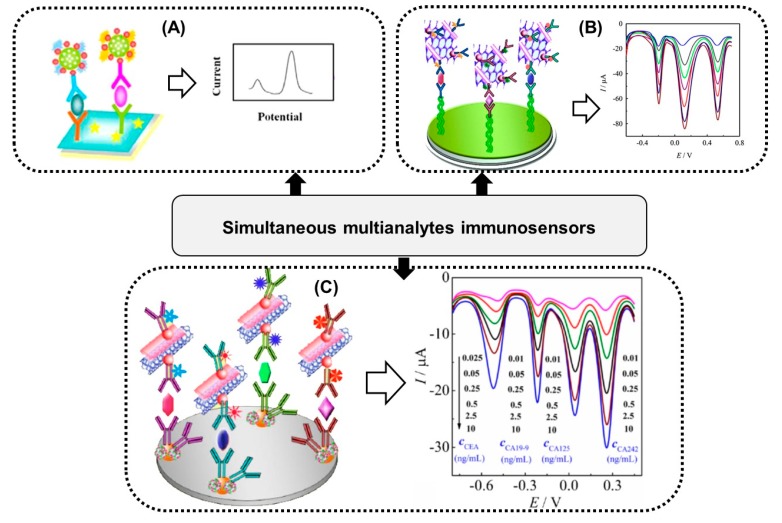
Schematic illustrations of immunosensors based on a multi-electroactive probe strategy. (**A**) simultaneous detection of two analytes using neutral red and thionine labels conjugated to specific antibodies (reprinted with permission from Wu et al. [63]). (**B**) Simultaneous detection of three analytes using thionine, ferrocenecarboxylic acid, and tris(2,2′-bipyridine-4,4′-dicarboxylic acid)cobalt(III) labels conjugated to specific antibodies (reprinted with permission from Zhu et al. [65]). (**C**) Simultaneous detection of four biomarkers using anthraquion-one 2-carboxylic acid, thionine, ferrocenecarboxylic acid, and tris(2,2′-bipyridine-4,4′-dicarboxylic acid)cobalt(III) labels conjugated to specific antibodies (reprinted with permission from Zhu et al. [67]).

In the first example paper (Figure 5A), a sandwich complex has been formed by composing two different primary antibodies immobilized onto reduced graphene oxide-tetraethylene pentamine and two antigens, carcinoembryonic and squamous cell carcinoma, as well as secondary antibodies labelled with two redox probes: neutral red and thionine [63]. This electrochemical immunosensor was proposed for the simultaneous determination of both antigens based on the peak current changes of neutral red and thionine before and after antigen-antibody reaction. In this case, EDC/NHS chemistry was used in order to conjugate secondary antibodies with different redox probes by the formation of an amide link between the redox probe and the carboxyl group of the antibody. The reproducibility of the sensor proposed was quite good with an RSD of 3.3% and 4.8% for both antigens. A similar approach has been recently described for simultaneous detection of two pathogens: Aeromonas hydrophila (Ah) and Pseudomonas aeruginosa (Ps) [64]. In sandwich format, thionine-labelled anti-Ah and ferrocene-labelled anti-Ps were used as redox probes, respectively. The composite platform based on a zeolitic imidazole framework/gold nanoparticle allowed for high loading of antibodies and thereby achieved a very low detection limit. The selectivity and specificity studies showed that the fabricated immunosensor was free from interferences. In the next work [65], Zhu and co-workers built a sandwich-type immunosensor for determination of three biomarkers as model analytes (Figure 5B). Three kinds of primary antibodies were immobilized onto protein A/Nafion modified glassy carbon electrode. Three secondary antibodies cross-linked with three redox probes, including thionine, ferrocenecarboxylic acid, and tris(2,2′-bipyridine-4,4′-dicarboxylic acid)cobalt(III), were integrated in functionalized graphene sheets containing gold nanoparticles (AuNPs) and carboxyl groups. The reductive peak current of three redox probes in differential pulse voltammetry were used as analytical signals quantitatively related to the concentration of each analyte. In another example of a sandwich immunosensor for simultaneous determination of three analytes, the same three redox species as above were used as signal tags and labelled to antibodies with gold nanoparticle-coated carbon nanotubes used as signal enhancers [66]. The simultaneous determination of four biomarkers on a single glassy carbon electrode has been presented by Zhu and co-workers [67]. Anthraquion-one 2-carboxylic acid, thionine, ferrocenecarboxylic acid and tris(2,2′-bipyridine-4,4′-dicarboxylic acid)cobalt(III) were applied as redox tags for labelling the detection of antibodies in a sandwich immunosensor format (Figure 5C). Well resolved peaks of all redox tags observed at potentials of −0.52, −0.21, 0.0 and 0.26, respectively were used for quantitative analysis of four antigens. The use of gold nanoparticles (AuNPs) and functionalized carbon nanotubes (CNTs) in the construction of this immunosensor caused low background current and excellent sensitivity and selectivity. The simultaneous detection of six biomarkers has been proposed [68] by combining a multi-label strategy and multi-spot assay with an array electrode. This electrode consisted of three detection spots and one control. Each detection spot was composed of two capture antibodies able to detect two appropriate proteins and two detection antibodies labelled with thionine and anthraquion-one 2-carboxylic acid.

## 6. Conclusions and Future Perspectives

A lot of effort has been made in the development of modern electrochemical immunosensors in the last few years. Due to advantages such as high selectivity, sensitivity, portability, the possibility of multi-analyte analysis, low cost, the opportunity for miniaturization and low sample requirements, these analytical devices have found increased attention of researchers in medicine, pharmaceutical and food industry fields. 

However, there is still an urgent need to improve features of electrochemical immunosensors by developing new concepts of antibody conjugation. Conjugates obtained by non-specific attachment can be more heterogeneous and variable. Significant effort should be made to develop site-specific methods used for obtaining homogenous conjugates. A proper conjugation method can guarantee sufficient interaction between antibodies and antigens. Lysines with free amines are less uniformly distributed in the antibody. Thus, the obtained product of conjugation could be less specific and possess fewer groups that are potentially useful for attachment to the surface of immunosensors. A possible alternative could be conjugation between reduced free thiol groups on the antibody with a redox active molecule containing the maleimide group. Furthermore, taking into account the advantages of the on-bead conjugation method, antibodies labelled with different redox active labels could be obtained maintaining the best functionality.

The unquestionable advantages of multiplexed immunosensors using a single electrode include simplicity, low cost and possibility to obtain an analytically useful signal in a single record. However, there are still problems arising from mutual interference or overlapping of the electrochemical signal (“cross-reactivity”). This challenge can be achieved by finding appropriate redox active labels which differ in electrochemical behavior when attached to antibodies on the same electrode platform. There is still a chance for improvement in designing new interfaces in order to obtain well separated redox peaks without overlapping each other. Moreover, there is still a chance for a novel multiplexed electrochemical immunosensor based on different distinguishable signals and tag-labelled antibodies performed using one-step immunoreaction. Such an approach, in comparison with sandwich-type immunosensors, can shorten the time of analysis, reduce the incubation time and reduce the cost of analysis.
